# Correction: High photocatalytic yield in the non-oxidative coupling of methane using a Pd–TiO_2_ nanomembrane gas flow-through reactor

**DOI:** 10.1039/d4ey90022g

**Published:** 2024-10-15

**Authors:** Victor Longo, Luana De Pasquale, Francesco Tavella, Mariam Barawi, Miguel Gomez-Mendoza, Víctor de la Peña O’Shea, Claudio Ampelli, Siglinda Perathoner, Gabriele Centi, Chiara Genovese

**Affiliations:** a Department of Chemical, Biological, Pharmaceutical and Environmental Sciences and CASPE (INSTM), University of Messina Viale F. Stagno D’Alcontres 31 98166 Messina Italy chiara.genovese@unime.it; b Photoactivated Processes Unit, IMDEA Energy Avda. Ramón de la Sagra, 3, Móstoles 28935 Madrid Spain

## Abstract

Correction for ‘High photocatalytic yield in the non-oxidative coupling of methane using a Pd–TiO_2_ nanomembrane gas flow-through reactor’ by Victor Longo *et al.*, *EES. Catal.*, 2024, **2**, 1164–1175, https://doi.org/10.1039/D4EY00112E.

Fig. 4(c) is incorrect in the original article. The complete Fig. 4 should appear as below.

**Fig. 4:**
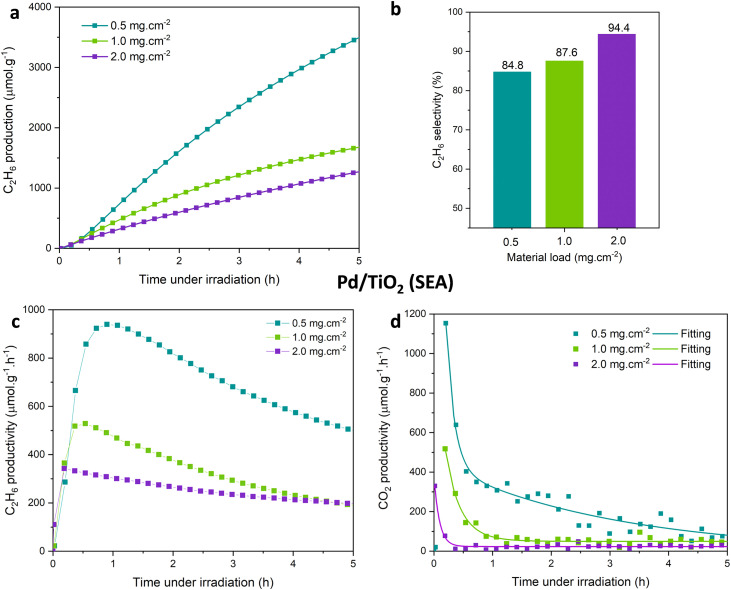
(a) Ethane yield (μmol g^−1^), (b) ethane selectivity (%), and (c) ethane and (d) CO_2_ production rates (μmol g^−1^ h^−1^) by photocatalytic NOCM over Pd/TiO_2_ (SEA) samples with loadings of 0.5, 1.0 and 2.0 mg cm^−2^. Reaction conditions as in **Fig. 2**.

The Royal Society of Chemistry apologises for these errors and any consequent inconvenience to authors and readers.

